# Good functional outcome but high rates of instability recurrence after posterior open‐wedge glenoid osteotomy for the treatment of posterior shoulder instability with increased glenoid retroversion at mid‐term follow‐up

**DOI:** 10.1002/ksa.12548

**Published:** 2024-12-15

**Authors:** Maximilian Hinz, Lorenz Fritsch, Sebastian Siebenlist, Lucca Lacheta, Jonas Pogorzelski, Marco‐Christopher Rupp, Bastian Scheiderer

**Affiliations:** ^1^ Department of Sports Orthopaedics Technical University of Munich Munich Germany

**Keywords:** autologous bone graft, glenoid osteotomy, joint‐preserving, posterior humeral head subluxation, salvage procedure, shoulder instability

## Abstract

**Purpose:**

To evaluate clinical, functional and radiological mid‐term outcomes following posterior open‐wedge glenoid osteotomy (POWGO) for the treatment of posterior shoulder instability (PSI) associated with increased glenoid retroversion.

**Methods:**

Patients who underwent POWGO for the treatment of symptomatic PSI with glenoid retroversion >10° and participated in a previous study assessing short‐term outcomes were included after a minimum follow‐up of 5 years. Clinical (Rowe score and physical examination) and functional outcomes (Oxford Shoulder Instability Score [OSIS] and visual analogue scale [VAS] for pain) were assessed. Preoperative versus follow‐up magnetic resonance imaging (MRI) assessments were compared for changes in posterior humeral head subluxation (PHHS) and progression of osteoarthritis (shoulder osteoarthritis severity [SOAS] score).

**Results:**

Eight patients (nine shoulders) were included 92.0 months (88.0–109.5 months) post‐operatively, of which seven patients (eight shoulders) underwent MRI. Shoulder function was good (Rowe score: 80.0 [76.3–91.3], OSIS: 41.0 [31.0–41.5]) and pain levels were low (VAS for pain: 3.0 [1.0–3.0]) at follow‐up. Overall, the degree of PHHS did not change between preoperatively and follow‐up (*p* > 0.05). Four shoulders demonstrated PHHS preoperatively, of which two had a centred humeral head at follow‐up. Shoulder osteoarthritis progressed significantly (SOAS score: 17.0 [11.0–24.5] to 33.0 [31.0–45.0], *p* = 0.018). Residual PSI was evident in 75.0% of shoulders.

**Conclusion:**

At mid‐term follow‐up, POWGO for PSI associated with increased glenoid retroversion led to good functional outcomes but failed to reliably restore posterior shoulder stability and prevent osteoarthritis progression.

**Level of Evidence:**

Level IV.

**Abbreviations**
MRI: magnetic resonance imaging
OSIS: Oxford Shoulder Instability Score
PHHS: posterior humeral head subluxation
POWGO: posterior open‐wedge glenoid osteotomy
PSI: posterior shoulder instability
ROM: range of motion
SOAS: shoulder osteoarthritis severity
VAS: visual analog scale

## INTRODUCTION

The aetiology of recurrent posterior shoulder instability (PSI) is multifactorial and includes trauma [15], muscle imbalances of the transverse force couple [22], a higher situated and more horizontally oriented acromion [2, 9, 12, 18, 21], an anteriorly situated glenoid vault [1], glenoid dysplasia [7] and increased glenoid retroversion [7, 10, 20, 25, 26, 27]. The latter is thought to be a major reason for posterior humeral head subluxation (PHHS) causing eccentric glenohumeral osteoarthritis [5].

Patients with PSI refractory to non‐operative treatment may undergo posterior capsulolabral repair. In the presence of increased glenoid retroversion; however, this procedure is associated with inferior outcomes and higher revision rates [15, 32]. These patients may benefit from undergoing a posterior open‐wedge glenoid osteotomy (POWGO), which corrects glenoid retroversion and potentially recenters the humeral [13]. Previous studies reported good functional outcomes, but with high rates of residual PSI, no reliable change in PHHS as well as a progression of glenohumeral osteoarthritis [6, 19, 28, 31]. Currently, outcome data beyond short‐term follow‐up are still scarce.

Therefore, the purpose of the present study was to evaluate clinical, functional and radiological mid‐term outcomes following POWGO for the treatment of PSI associated with increased glenoid retroversion. It was hypothesized that an overall good functional outcome would be observed, but that a high rate of residual PSI, no change in PHHS as well as a significant progression of osteoarthritis would be observed. It was hypothesized that POWGO would lead to a good functional outcome but would not restore posterior shoulder stability or prevent osteoarthritis progression.

## MATERIALS AND METHODS

The present study received approval from the institutional review board of the Technical University of Munich (reference: 290/17 S) and was conducted according to the Declaration of Helsinki. All patients signed written and informed consent forms. Patients who underwent POWGO for the treatment of PSI associated with increased glenoid retroversion (>10°) between February 2009 and August 2016 and participated in a previous study investigating short‐term outcomes (>12 months) [17] were followed up after a minimum of 5 years.

### Surgical technique

**Figure 1:**
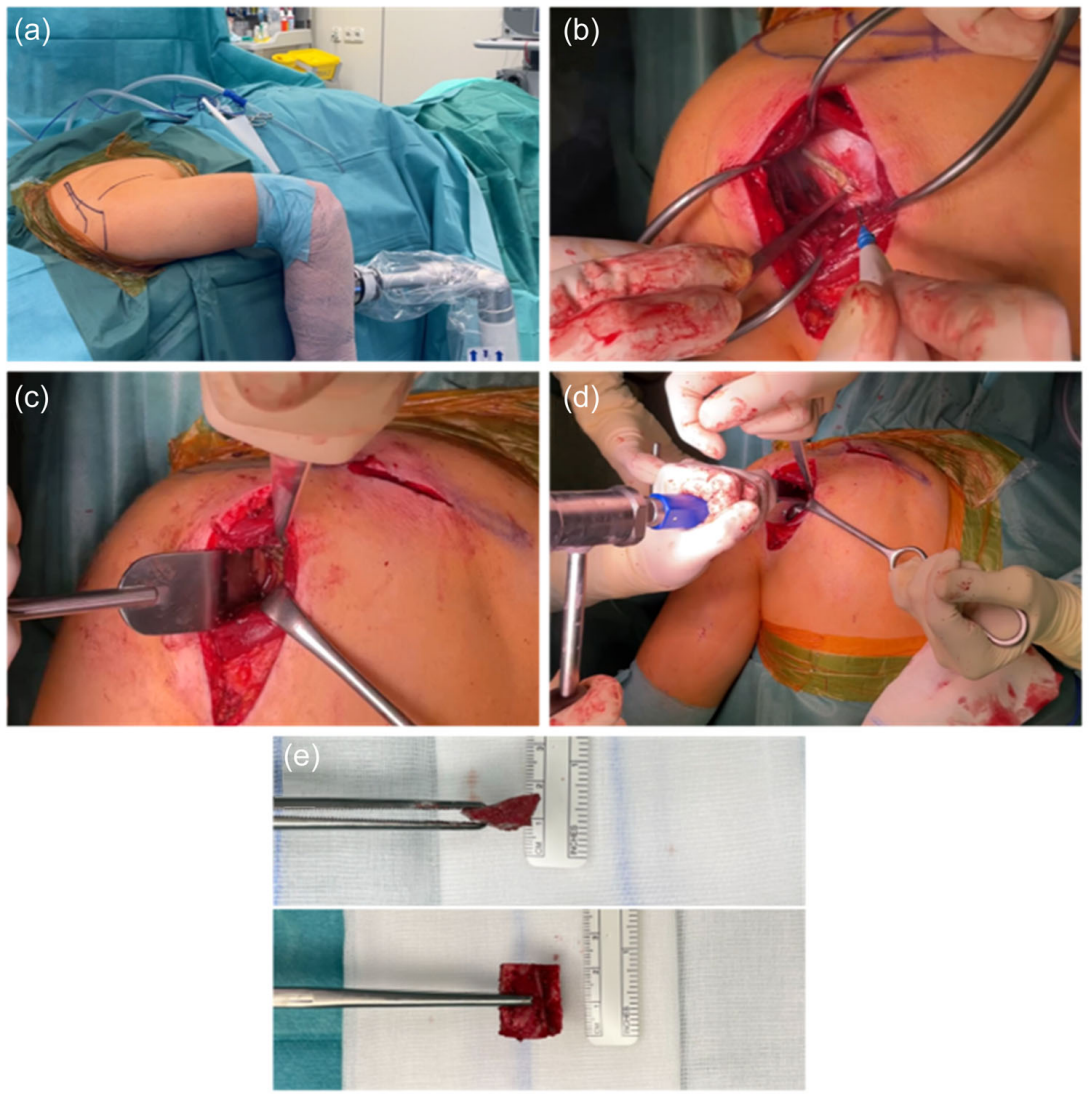
Surgical technique. (a) The patient was placed in the prone position. (b) The infraspinatus muscle was split along its fibres. (c) A blunt retractor was introduced into the glenohumeral joint. (d) The osteotomy was performed 10 mm medial to the posterior glenoid rim. (e) A wedge‐shaped autologous bone graft from the scapular spine was prepared on a side table.

The surgical technique used has previously been described [17]. Patients were placed in the prone position (see Figure 1a). A posterior deltoid‐splitting approach was used to expose the underlying infraspinatus muscle, which was split along its fibres (see Figure 1b). A T‐shaped incision of the posterior capsule was established, and a blunt retractor was introduced into the glenohumeral joint (see Figure 1c). The osteotomy was performed 10 mm medial to the posterior glenoid rim (see Figure 1d). A wedge‐shaped autologous bone graft, as shown in Figure 1e, either harvested from the scapular spine or the iliac crest, was then impacted. Finally, a T‐shaped shift of the posterior capsule was performed.

### Post‐operative rehabilitation

For the first 6 post‐operative weeks, the operated shoulder was immobilized in a shoulder brace (medi SLK 90, medi GmbH & Co. KG) with the arm in neutral rotation and 5° of abduction. During this time, passive and active‐assisted range of motion (ROM) were allowed, with shoulder flexion limited to 60° and internal limitation limited to 45°. Clearance for return to activity was given 6 months post‐operatively.

### Clinical and functional outcome assessment

Shoulder function (Rowe score, Oxford Shoulder Instability Score [OSIS]) and pain (visual analogue scale [VAS] for pain) were evaluated. A standardized physical examination was performed to assess active shoulder ROM and posterior instability using the Jerk test [16] and the Kim test [16]. These tests were rated positive if patients reported pain, apprehension or if a subluxation/dislocation occurred during testing. Additionally, PSI recurrence, defined as a positive Jerk and/or Kim test, a post‐operative instability event or PHHS, and revision rates during follow‐up were recorded.

### Radiological outcome assessment

Magnetic resonance imaging (MRI) was performed at follow‐up and compared to preoperative MRI. The degree of PHHS was calculated using the adapted subluxation index as described by Gerber et al. (centred head: 35%–65%; posterior subluxation: >65%, anterior subluxation: <35%) [8]. Shoulder osteoarthritis was quantified with the shoulder osteoarthritis severity (SOAS) score [14]. The SOAS score is a semiquantitative MRI‐based score that evaluates osteoarthritic changes and their progression with higher values indicating greater global shoulder osteoarthritis. The score has been successfully utilized to quantify osteoarthritic changes in patients who underwent joint‐preserving procedures, such as rotator cuff repair [4] or superior capsular reconstruction [11].

### Statistical analysis

Data were analyzed using SPSS 26.0 (IBM‐SPSS). Categorical variables are presented in counts and percentages. Continuous variables are shown as median (25%–75% interquartile range). For group comparisons of continuous variables, the Wilcoxon rank‐sum test was applied. Statistical significance was set at a *p* value of <0.05. Due to the rarity of this injury and the prerequisite of participating in the previous study assessing short‐term outcomes, all eligible patients were considered and a power analysis was not performed.

## RESULTS

**Figure 2:**
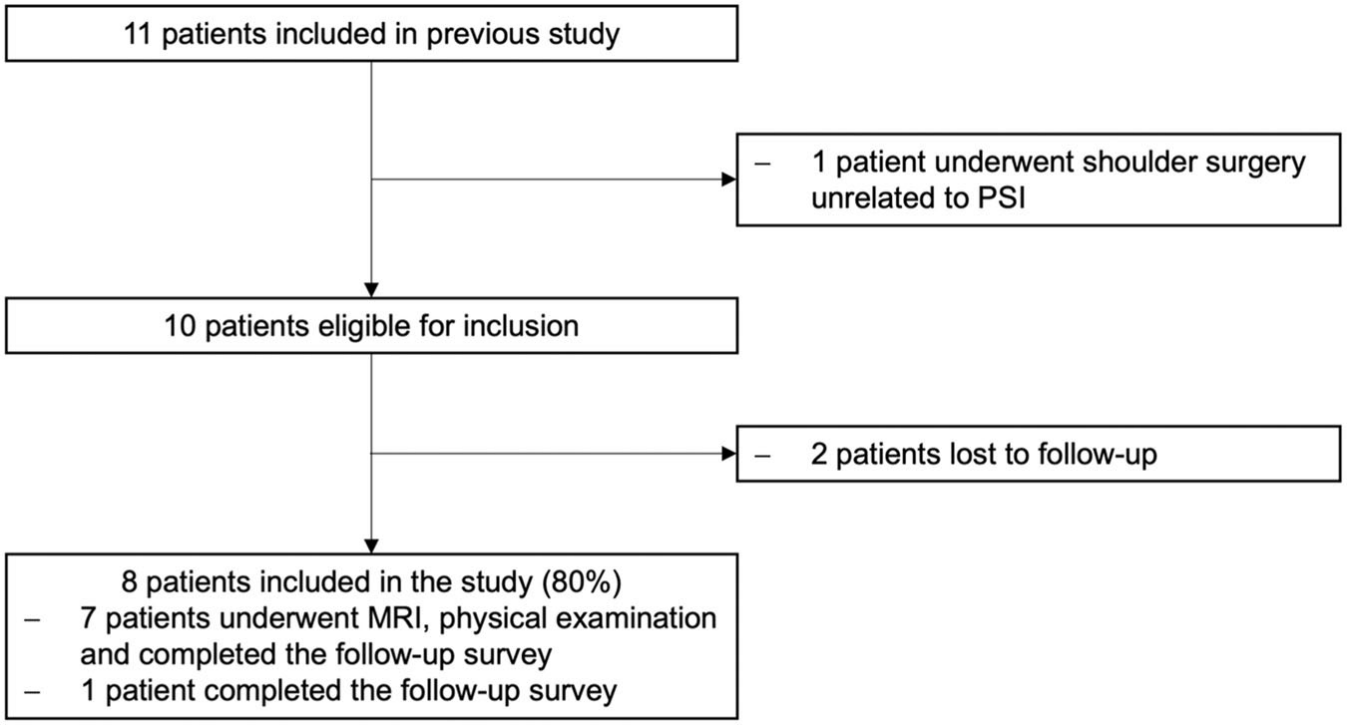
Flowchart for study inclusion. MRI, magnetic resonance imaging, PSI, posterior shoulder instability.

Eight patients (nine shoulders; 80.0% follow‐up) were available for follow‐up after a median of 92.0 months (88.0–109.5 months) post‐operatively (see Figure 2). All patients were male. The median age at the time of surgery was 26.0 years (19.0–31.0 years).

### Clinical and functional outcome

Shoulder function was good at mid‐term follow‐up and did not significantly differ from short‐term follow‐up (Rowe score: 87.5 [77.5–97.5] to 80.0 [76.3–91.3], *p* > 0.05; OSIS: 42.5 [29.0–44.0] to 41.0 [31.0–41.5], *p* > 0.05). Post‐operative pain was mild (VAS for pain: 3.0 [1.0–3.0]). Active shoulder flexion (operated shoulder: 170.0° [150.0–177.5°] vs. contralateral shoulder: 170° [170.0–177.5°]), abduction (operated shoulder: 180.0° [122.5–180.0°] vs. contralateral shoulder: 180° [172.5–180.0°]) and external rotation (in 90° of abduction) ROM (operated shoulder: 80.0° [80.0–90.0°] vs. contralateral shoulder: 90.0° [80.0–90.0°]) did not differ significantly from the contralateral side (*p* > 0.05). Of the eight shoulders that were physically examined, five (65.5%) showed PSI with a positive Jerk and/or Kim test. One patient (12.5%) reported two posterior shoulder subluxations post‐operatively. None of the patients underwent revision surgery by follow‐up.

### Radiological outcome

Overall, the degree of PHHS did not change between preoperatively and follow‐up (69 [61–73]% to 71 [54–72]%, *p* > 0.05). Of the eight shoulders that underwent MRI at follow‐up, four shoulders (50.0%) showed PHHS preoperatively, of which two shoulders had a centred humeral head at follow‐up. Four shoulders (50.0%) demonstrated a centred humeral head preoperatively, of which two developed PHHS by follow‐up. Overall, shoulder osteoarthritis progressed significantly from preoperatively to follow‐up (SOAS score: 17.0 [11.0–24.5] to 33.0 [31.0–45.0], *p* = 0.018).

### Shoulder instability

At follow‐up, six out of eight shoulders (75.0%) that underwent both physical examination and MRI showed signs of PSI (positive Jerk and/or Kim test: 62.5% [five out of eight], post‐operative instability event: 12.5% [one out of eight], PHHS: 50.0% [four out of eight]).

## DISCUSSION

The most important finding of the present study was that despite the favourable functional outcomes and mild pain levels observed at mid‐term follow‐up, 75.0% of patients who underwent POWGO demonstrated signs of residual PSI. Additionally, a significant progression of osteoarthritis was observed.

Satisfactory functional outcomes, but high rates of residual PSI, were also reported in recent systematic reviews analyzing POWGO as a treatment for PSI associated with increased glenoid retroversion [19, 28]. Specifically, in patients with preoperative PHHS, POWGO may not reliably restore humeral head centration [24], which was observed both in the present study and in a long‐term study by Waltenspül et al. [31]. Subsequently, the same authors proposed a combined POWGO and glenoid concavity reconstruction using a J‐shaped iliac crest autograft whereby both glenoid retroversion and glenoid dysplasia were addressed. With this technique, an overall reduction of PHHS as well as restitution of humeral head centration was achieved in half of the patients with preoperative PHHS at short‐term follow‐up [6]. Recently, Moroder et al. [23] described the arthroscopic posterior articular coverage and shift procedure as an alternative to POWGO. Similar to POWGO, a significant improvement in shoulder function was observed at short‐term follow‐up, but the procedure failed to restitute humeral head centration [23]. Gerber et al. [9] proposed the scapular (acromion and glenoid) corrective osteotomy for posterior escape procedure that, besides addressing the glenoid, also changes the morphology of a high and horizontally oriented acromion, which has been linked to the onset of PSI in the recent literature [2, 9, 12, 18, 21]. The authors reported an excellent outcome at 2 years post‐operatively in a single patient, but data with longer follow‐up and in a larger cohort were not available [9]. To date, the optimal treatment for PSI with PHHS is unknown. This may be related to the fact that PHHS, beyond glenoid retroversion and glenoid dysplasia, is co‐influenced by other factors. These may include an anteriorly positioned glenoid vault [1] and muscle imbalances of the transverse force couple [22], both of which may persist following POWGO. Additionally, the relationship between PHHS and glenoid retroversion is not yet fully understood [5, 26, 29].

Besides PSI, increased glenoid retroversion and PHHS may lead to early‐onset eccentric glenohumeral osteoarthritis [1, 5, 30]. Ortmaier et al. [24] analyzed the effect that POWGO had on the progression of osteoarthritis in young patients with glenohumeral osteoarthritis associated with increased glenoid retroversion and PHHS. Glenoid retroversion was reduced significantly, but PHHS persisted and progression of osteoarthritis was observed in 20% of the shoulders. Progression of glenohumeral osteoarthritis following POWGO for the treatment of PSI was also reported in previous studies [6, 31].

The present study has several limitations that should be mentioned. First, the small number of patients is notable; however, this is attributable to the rarity of this pathology and is comparable to other studies on this topic [6, 31]. Second, PHHS was assessed via MRI, whereas it was assessed via computed tomography in several other studies [6, 31], which may have confounded the results. Nonetheless, this should only have a limited effect on the comparability of the data as both MRI and computer tomography show a similar efficacy for measuring PHHS [3].

## CONCLUSION

At mid‐term follow‐up, POWGO for PSI associated with increased glenoid retroversion led to good functional outcomes but failed to reliably restore shoulder posterior stability and prevent osteoarthritis progression.

## AUTHOR CONTRIBUTIONS

Maximilian Hinz, Lorenz Fritsch, Lucca Lacheta, Jonas Pogorzelski and Bastian Scheiderer designed the study. Maximilian Hinz and Lorenz Fritsch collected the data. Maximilian Hinz performed the statistical analysis. Maximilian Hinz and Lorenz Fritsch wrote the manuscript. Marco‐Christopher Rupp and Sebastian Siebenlist supported data interpretation and critically reviewed the manuscript. All authors read and approved the final manuscript.

## CONFLICT OF INTEREST STATEMENT

Maximilian Hinz, Lorenz Fritsch, Lucca Lacheta, Jonas Pogorzelski and Marco‐Christopher Rupp declare no conflict of interest. Sebastian Siebenlist has received consultant fee payments from Arthrex GmbH, KLS Martin Group and medi GmbH & Co. KG unrelated to this study. Bastian Scheiderer has received consultant fee payments from Arthrex GmbH unrelated to this study.

## ETHICS STATEMENT

The study was approved by the institutional review board of the Technical University of Munich (reference: 290/17 S). Written informed consent was obtained from all patients.

## Data Availability

The data that support the findings of this study are available on request from the corresponding author. The data are not publicly available due to privacy or ethical restrictions.
